# Reviews of fungi and mycotoxins in Chinese dark tea

**DOI:** 10.3389/fmicb.2023.1120659

**Published:** 2023-02-22

**Authors:** Wei Xu, Yi-qiao Zhao, Wen-bao Jia, Si-yu Liao, Tunyaluk Bouphun, Yao Zou

**Affiliations:** ^1^College of Horticulture, Tea Refining and Innovation Key Laboratory of Sichuan Province, Sichuan Agricultural University, Chengdu, China; ^2^Faculty of Science and Agricultural Technology, Rajamangala University of Technology Lanna Lampang, Lampang, Thailand

**Keywords:** dark tea, mycotoxins, fungi, contamination, masked mycotoxins

## Abstract

The fermentation is the main process to form the unique flavor and health benefits of dark tea. Numerous studies have indicated that the microorganisms play a significant part in the fermentation process of dark tea. Dark tea has the quality of “The unique flavor grows over time,” but unscientific storage of dark tea might cause infestation of harmful microorganisms, thereby resulting in the remaining of fungi toxins. Mycotoxins are regarded as the main contributor to the quality of dark tea, and its potential mycotoxin risk has attracted people’s attention. This study reviews common and potential mycotoxins in dark tea and discusses the possible types of masked mycotoxins in dark tea. A summary of the potential risks of mycotoxins and masked mycotoxins in dark tea is presented, intending to provide a reference for the prevention and risk assessment of harmful fungi in dark tea.

## 1. Introduction

Dark tea, one of the six major tea groups, is a post-fermented tea that is produced by solid-state fermentation involving microorganisms (Wang et al., 1991a). In recent years, it has been proven that dark tea produces many specific products that are not found in other teas during processing, for which the damp-heat effect and microbial extracellular enzymes are accountable (Wang et al., 1991b; Qi et al., 2004; Chen and Qi, 2010). Different kinds of dark tea have been found to have their own dominant fungal strains during the processes of fermentation and storage (Xiong, 2017), and parts of these strains can synthesize fungal toxins.

**Figure 1:**
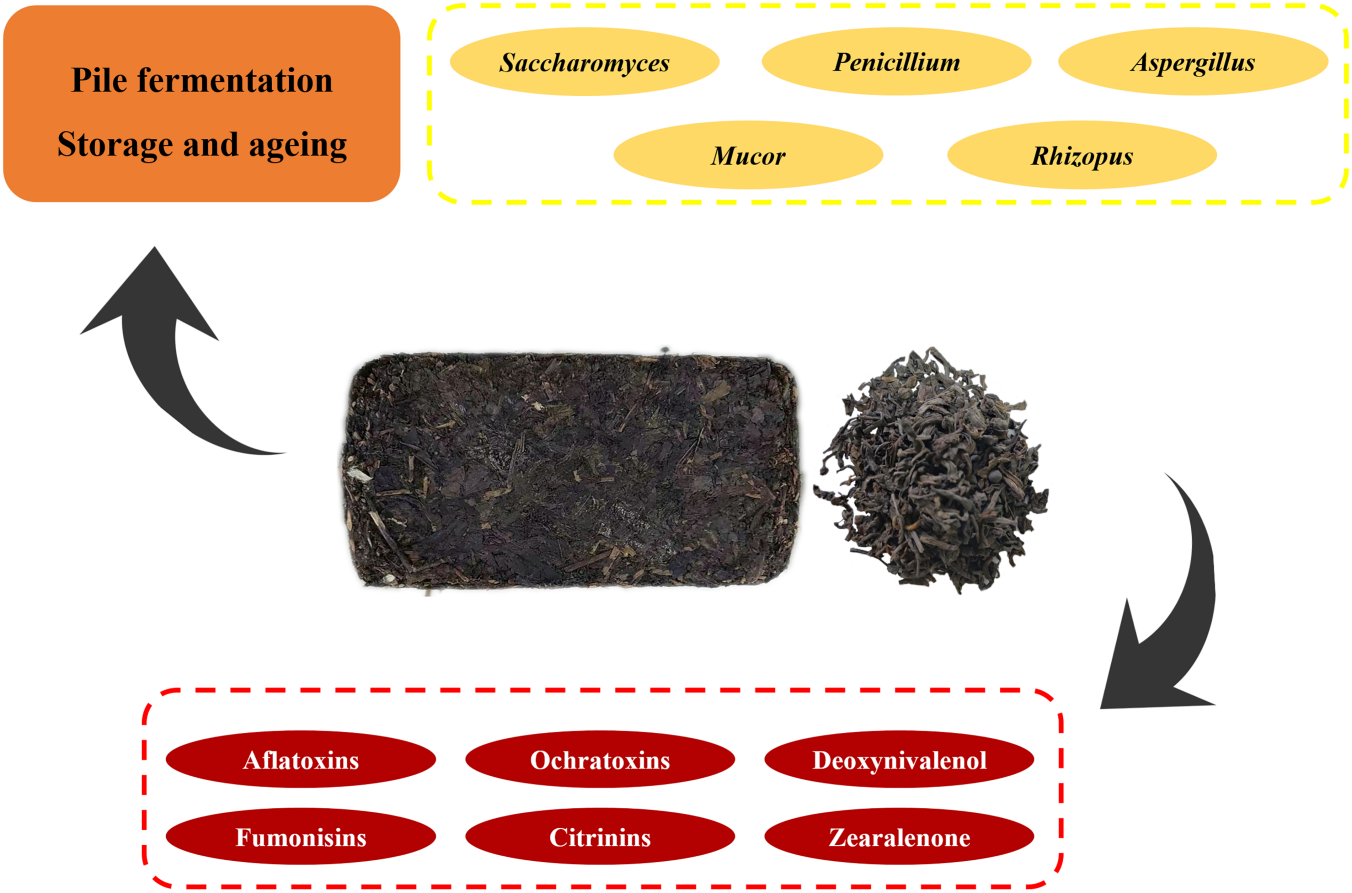
The main dominant microorganisms and potentially contaminating mycotoxins in dark tea.

Mycotoxins widely existed in all kinds of food, which is a serious threat to human health and safety, which has become one of the most important issues in the field of food safety (Bhat et al., 2010; Medina et al., 2015, 2017; Haque et al., 2020; Martínez-Culebras et al., 2021; Zhang et al., 2021). Mycotoxins are secondary metabolites produced by fungi that may be harmful to humans when ingested, inhaled, or in contact with skin (Marin et al., 2013). Fungi of many genera produce fungal toxins, mainly *Aspergillus*, *Penicillium*, *Alternaria*, *Fusarium*, and *Claviceps* genus (Murugesan et al., 2015; Jiang et al., 2018; Wang G. et al., 2020). The common fungal toxins are aflatoxins (AFTs), ochratoxins (OTs), deoxynivalenol (DON), fumonisins (FUMs), zearalenone (ZEN), patulin (PAT), and Citrinins (CITs) (Haque et al., 2020; Vargas Medina et al., 2021). There are also masked mycotoxins that have been overlooked in previous testing and assessment (Tan et al., 2016). The main dominant microorganisms and potentially contaminating mycotoxins in dark tea are shown in Figure 1. Recent evidence suggests that mycotoxins were detected in fermented tea (Liu et al., 2016; Mo et al., 2016; Hu et al., 2019; Xu W. et al., 2019), which indicated that there is indeed a possibility that dark tea is contaminated with mycotoxins during the production and processing as well as storage processes.

There exist several methods for measuring the mycotoxins, for instance: thin-layer chromatography (TIC)(Zhao et al., 2016), enzyme-linked immunosorbent assay (ELISA)(Li et al., 2016; Zhang, 2017; Zhu et al., 2017), high performance liquid chromatography (HPLC) (Kong et al., 2014; Li and He, 2020; Iqbal et al., 2021), liquid chromatography–tandem mass spectrometry (LC–MS/MS) (Kresse et al., 2019; York et al., 2020), and gas chromatography (GC) (Li et al., 2000; Lin et al., 2013). The currently existing measuring methods of mycotoxins have trouble detecting the masked mycotoxins in dark tea (Tan et al., 2016). Therefore, we need to pay more attention to the safety of dark tea during production and storage to ensure the health and safety of consumers. This paper reviews the contamination status and contamination levels of common mycotoxin species in dark tea, also speculates on the masked mycotoxins that may exist in dark tea, and discusses the main microbial sources of mycotoxin production in dark tea and the possible ways to cause mycotoxin contamination processes in dark tea, which may provide a reference for food safety policymakers and researchers.

## 2. Common fungal toxins in dark tea

### 2.1. Aflatoxin

Aflatoxins (AFTs) are a class of secondary metabolites produced by *Aspergillus parasiticus* and *Aspergillus flavus* (Piekkola et al., 2012). Aflatoxins are readily produced by *Aspergillus flavus* under relatively high air humidity and temperature conditions (Yu et al., 2004). Aflatoxins can be classified into several types, such as B_1_, B_2_, G_1_, and G_2_. Among them, aflatoxin B_1_ (AFB_1_) is the most toxic and has been classified as a class I carcinogen by the World Health Organization Cancer Research Unit (Yogendrarajah et al., 2015).

Currently, numerous studies have indicated that there is a risk of aflatoxins in dark tea. Liu et al. (2016) used high performance liquid chromatography–tandem mass spectrometry (HPLC–MS) for testing 10 samples of Pu-erh tea, among which AFB_1_ was detected in two samples at 3.1 μg/kg and 7.5 μg/kg, respectively. Wu J. (2013) used HPLC to detect AFB_1_ in seven samples at concentrations greater than 5 μg/kg in 60 samples of Pu-erh tea. Cui et al. (2020) examined 158 dark tea samples by liquid chromatography-mass spectrometry, two of which tested positive for aflatoxins. One Kangzhuan tea contained 2.07 μg/kg of AFB_1_; the other Fuzhuan tea contained four aflatoxins at concentrations of AFB_1_: 1.24 μg/kg, AFB_2_: 0.78 μg/kg, AFG_1_: 0.81 μg/kg, AFG_2_: 1.04 μg/kg. The previous reports have shown that dark tea has the possibility of being contaminated with aflatoxins, but levels and detection rates of aflatoxins are very low, so the risk exposure level of aflatoxin in dark tea is very low. Aflatoxin, as a highly toxic carcinogen, has always been a great concern for tea consumers. Therefore, the most effectively way to prevent dark tea from being contaminated by exogenous aflatoxin during processing and storage is an issue worthy of attention and consideration.

### 2.2. Ochratoxins

Ochratoxin (OTs) is a group of secondary metabolites produced by fungi *Aspergillus* and *Penicillium* (Wang et al., 2018). Ochratoxins were first isolated by Vander Merwe from the metabolites of *Aspergillus ochraceus* (Van der Merwe et al., 1965). Ochratoxin is classified into several types based on its structure, including A, B, C, and, with ochratoxin A (OTA) ranking first. Available studies have demonstrated that OTA is nephrotoxic, hepatotoxic, immunotoxic, genotoxic, neurotoxic, and teratogenic (Chen et al., 2018). It is classified by the International Agency for Research on Cancer (IARC) as a renal carcinogen in animals and a probable carcinogen in humans. OTA contaminates a variety of crops and teas (Zhang, 2017; Deng et al., 2020; He et al., 2020; Zhao et al., 2022), with the main OTA-producing fungi differing depending on environmental conditions. The main fungi that produce OTA are shown in Table 1 (Sánchez-Hervás et al., 2008; Iqbal et al., 2014; Wang et al., 2016; Deng et al., 2020). Only parts of some strains of *Aspergillus niger* have the ability to produce OTA. There are differences in the ability to synthesize OTA on different substrates and under different environmental conditions (Deng et al., 2020).

**Table 1:** The main fungi producing OTA.

Genus	Species
*Aspergillus*	*A. niger, A. carbonarius, A. ochraceus, A. affinis, A. terreus, A. fumigatus, A. versicolor, A. tubingensis, A. petrakii, A. sclerotiorum, A. westerdii. A. tubingensis, A. petrakii, A. sclerotiorum, A. westerdijkiae, A. alliaceus, A. sulphureus, A. melleu, A. parasiticus, A. sulphureus, A. melleu, A. sulphureus. A. parasiticus, A. welwitschiae*
*Penicillium*	*P. verrucosum, P. variabile, P. nordicum, P. cyclopium, P. chrysogenum, P. polonicum, P. viridicatum*

Many researchers have tested OTA in dark tea using various testing techniques, and the results are detailed in Table 2. Combined with the above results, it can be observed that OTA in dark tea has a certain exposure risk. Many *Aspergillus* spp. fungi are dominant in the processing of dark tea, which is closely related to the formation of dark tea sensory quality (Wu J., 2013; Xiong, 2017). On the other hand, Fungi of the genus *Penicillium* have been detected during the storage of dark tea (Haas et al., 2013). OTA-producing fungi have similar strains to the dominant fungi in dark tea solid-state fermentation and storage As a result, there is a risk of OTA exposure during both the processing and storage of dark tea. The dominant strains in dark tea during the solid-state fermentation process are similar to many OTA-producing strains, but OTA is not only present in dark tea samples. OTA has been found in black and green tea samples, according to some studies (Santos et al., 2009; Malir et al., 2014). This indicates that the source of OTA in dark tea has two possibilities, therefore we should pay much more attention to fungal production of toxicity and exogenous contamination. And Zhao Z. et al. (2021) and Zhou et al. (2015a,b) isolated OTA-producing strains from dark tea samples.

**Table 2:** OTA detection in dark tea samples.

Dark tea types	Method	Positive rate	Contents (μg/kg)	References
Wet storage fermented dark tea	ELISA	100%	Less than 50	Liu et al. (2011)
Pu-erh Tea	HPLC	11%	0.65–94.7	Haas et al. (2013)
Pu-erh tea, etc.	HPLC–MS/MS	4%	0.22–0.44	Mo et al. (2016)
Pu-erh tea, Fu tea, etc.	UPLC–MS /MS	4%	4.2	Liu et al. (2016)
Pu-erh tea, etc.	UPLC-MS /MS	8%	0.9–6.7	Liu et al. (2017)
Pu-erh Tea	LC-MS/MS	0%	ND	Hu et al. (2019)
Pu-erh tea, Liupao tea, etc.	HPLC	1.85%	34.9–36.8	Ye et al. (2020)
Dark tea	HPLC	9.2%	2.51–12.62	Zhao et al. (2022)

### 2.3. Citrinin

Citrinin (CIT) was originally isolated from cultures of *Penicillium citrinum* by Hetherington and Raistrick (1931). CIT is a well-known hepatorenal toxin that causes functional and structural kidney damage as well as debilitation of liver metabolism (Flajs and Peraica, 2009; Vidal et al., 2018). In recent years, CIT has received much attention due to its toxic effects on mammals and its widespread presence in foods, where it is usually found together with OTAs (Silva et al., 2021). The main CIT-producing fungi are similar to the OTA-producing ones, mainly *Penicillium* and *Aspergillus* fungi (Sengling Cebin Coppa et al., 2019).

The kidney is the main target organ for the toxic effects of CIT, and there are also reports of CIT effects on the liver and bone marrow (Magan and Olsen, 2004). CIT is a fungal toxin widely present in food, and there is a possibility that dark tea is contaminated with CIT. Li and He (2020) examined 113 tea samples of Liupao tea from different years by HPLC, 37 positive CIT samples with a CIT content of 7.8–206.1 μg/kg were detected. Zhou et al. (2015a,b) isolated an OTA-producing *Penicillium chrysogenum* dominant strain from Pu-erh tea by inoculating it on raw dark tea with 35% moisture content at 30°C for 25 days, and the CIT content was 26.30 μg/kg.

Since the majority of OTA-producing fungi can produce CIT (Silva et al., 2021), OTA was also detected in many tea samples, which forced us to consider whether OTA-producing fungi in dark tea will also produce CIT. Bugno et al. (2006) isolated 10 genera of molds from herbs, 89.9% of the fungi were *Aspergillus* and *Penicillium* spp., and 21.97% of both *Aspergillus* and *Penicillium* spp. were able to produce CIT. Two hundred and sixty strains of *Penicillium* spp. were isolated from cereals and fruits by Andersen et al. (2004), and 85% of *Penicillium* spp. were able to produce CIT. In recent years, many other researchers have isolated and identified *Penicillium citrinum* from dark tea. Haas et al. (2013) isolated and identified two strains of *Penicillium citrinum* from traditional Pu-erh tea loose tea, three strains in organic Pu-erh tea loose tea, and three strains in tightly pressed Pu-erh tea, respectively. Su (2018) isolated and identified 34 fungal strains from four commercially available dark tea samples, including four strains of *Penicillium oryzae*. Zhao (2012) and Hu et al. (2020) isolated and identified *Penicillium citrinum* from ‘flowering’ of Fuzhuan tea and finished dark tea, respectively. *Aspergillus* spp. and *Penicillium* spp. fungi are very common in the production and processing of dark tea. *Penicillium citrinum* is also more common in dark tea. Therefore, there is a certain risk exposure level for CIT in dark tea, which poses potential health hazards to consumers. There are relatively few studies on CIT in dark tea samples, and there is almost no standard limit on the amount of CIT in dark tea.

### 2.4. Deoxynivalenol

Deoxynivalenol (DON), also known as vomitoxin, is named for its ability to cause vomiting in pigs. This is a secondary metabolite produced by *toxigenic Fusarium* species and others, combined with the high rate of contamination in both cereals and their products (Chen et al., 2017; Csikós et al., 2020; Wang and Wang, 2021; Yang et al., 2021). DON is potentially harmful to both humans and animals when it enters the food chain, such as causing loss of appetite, immunosuppression, nausea, and vomiting, and has been classified as a Class III carcinogen by the European Union (Pereira et al., 2019).

Although DON is mostly detected in cereals and their products, the researchers have not paid sufficient attention to it. In recent years, it has been reported that tea also has the possibility of being contaminated by DON. Yan (2019) examined 61 fermented dark tea samples, and DON was detected in three samples, including one Pu-erh tea with 8.6 μg/kg and two Hunan dark tea with 70.1 and 299.5 μg/kg, respectively. Hu et al. (2019) examined 174 Pu-erh tea samples, DON was detected in 30.63% of the samples. Although the content of DON in the detected dark tea samples is far below the limit, we should pay much more attention to DON, which can protect the health of consumers and promote the sustainable development of dark tea.

### 2.5. Masked mycotoxins

Masked mycotoxins, usually conjugated mycotoxins, are mycotoxin derivatives formed by microbial transformation, degradation, hydrolysis, reduction, glycosylation, formylation, etc. Most masked mycotoxins are less toxic or non-toxic than their original mycotoxins. However, some masked mycotoxins are not completely transformed and degraded, and under some specific conditions, these masked mycotoxins can be reconverted to mycotoxins under certain conditions (Berthiller et al., 2013; Tan et al., 2016).

The detection of masked mycotoxins is relatively cumbersome. In the initial studies, the “indirect method” was usually used as a quantitative method to detect the amount of masked mycotoxins. With the constant development of analytical techniques, the direct detection of masked mycotoxins has become more common. Currently, commonly used direct detection methods include LC–MS/MS and ELISA. LC–MS/MS method has been widely used for the detection of mycotoxin residues in cereals, grains, oils, and other foods for its high selectivity and sensitivity (Lozowicka et al., 2022). LC–MS/MS can simultaneously detect different components in mixed samples, so that masked mycotoxins, their prototypes, metabolites, and even different types of mycotoxins can be quantified simultaneously. Vendl et al. (2009) used LC–MS/MS to simultaneously quantify DON and ZON prototypes and their eight masked mycotoxins in cereal foods. Due to the shortage of commercial standards, studies on the quantitative analysis of masked mycotoxins are significantly limited. However, ELISA may respond to masked mycotoxins (Berthiller et al., 2013). The mycotoxins detection studies in tea, where results of the test by ELISA were mostly positive for mycotoxins (Santos et al., 2009; Liu, 2011), also forced us to think, whether there are also masked mycotoxins in tea.

Also, the detection of mycotoxins exposure levels in dark tea is mainly for their prototypes and little attention has been paid to the risk of masked mycotoxins. There are only a few reports on the preparation of standards, anabolic pathways of masked mycotoxins (Tan et al., 2016), which is the reason that masked mycotoxins deserve further attention and research. The common masked mycotoxins are masked trichothecenes, masked ZEN, masked fumonisins, masked OTA etc. Table 3 shows the common masked mycotoxins in naturally contaminated foods.

**Table 3:** Common masked mycotoxins.

Classify	Masked mycotoxin	Contaminated food	References
Masked trichothecenes	DON-3-glucoside	Wheat; malts; maize	Savard (1991)
	DON-3-glucoside; DON-15-glucoside	Wheat; maize	Berthiller et al. (2005)
	DON-3-glucoside	Wheat	Kluger et al. (2015)
	DON-3-glucoside	Beer	Kostelanska et al. (2009)
	3-AcDON; 15-AcDON; DON-3-glucoside	Wheat	Palacios et al. (2017)
	T-2 toxin 3-O-glucoside; HT-2 toxin 3-O-glucoside	Wheat; oat	Busman et al. (2011)
	T-2 toxin-di-glucoside; HT-2 toxin-di-glucoside	Corn powder	Nakagawa et al. (2013)
	T2-3-glucoside; HT2-3-glucoside; HT2-4-glucoside	Wheat; oats	Lattanzio et al. (2012)
Masked ZEN	Z14G	Wheat	Schneweis et al. (2002)
	Z14S	Wheat flour; bran flakes	Vendl et al. (2010)
	α-ZEL; β-ZEL; Z14G; α-ZELG; β-ZELG; Z14S	Wheat; maize; wheat; oats etc	De Boevre et al. (2012)
Masked fumonisins	HFB1	Corn flakes	Kim et al. (2003)
	HFBs	Maize	Dall Asta et al. (2009)

In addition to these masked mycotoxins detected in naturally contaminated food, there are also masked mycotoxins that have been identified but not yet observed in naturally contaminated food. All of the prototypes of mycotoxins mentioned above have been found in dark tea. Whether it forms masked mycotoxins in the same condition. Whether the masked mycotoxins are harmful to consumers. These questions force us to investigate more in the future.

## 3. Microbial sources of fugal toxins in Chinese dark tea

Dark tea belongs to the post-fermented tea category, and microorganisms participate and play an equally important role in its production and processing. Many researchers have isolated and identified the dominant microorganisms from the production and processing of dark tea as well as from the finished tea, and the more common dominant microbial genera are shown in Table 4.

**Table 4:** Common dominant genus in dark tea.

Type of dark tea	Dominant genus	References
Kangzhuan tea	*Aspergillus, Penicillium, Saccharomyces*	Fu and Guinian (2008)
Kangzhuan tea	*Aspergillus, Mucor, Mycobacterium, Rhizopus*	Xu (2010)
Kangzhuan tea	*Aspergillus, Penicillium, Saccharomyces*	Zheng (2013)
Sichuan dark tea	*Aspergillus, Penicillium, Saccharomyces, Rhizopus*	Jiang (2012b)
Ya’an Tibetan tea	*Aspergillus, Penicillium, Rhizopus*	Li (2019)
Pu-erh tea	*Aspergillus, Penicillium, Saccharomyces, Rhizopus*	Zhou et al. (2004)
Pu-erh tea	*Aspergillus, Saccharomyces*	Dong et al. (2009)
Pu-erh tea	*Aspergillus, Arxula*	Wang Q. et al. (2020)
Pu-erh tea	*Aspergillus, Penicillium, Saccharomyces*	Bi et al. (2014)
Pu-erh tea	*Aspergillus, Penicillium, Saccharomyces*	Zhang et al. (2012)
Pu-erh tea	*Aspergillus, Saccharomyces*	Hu (2013)
Pu-erh tea	*Aspergillus, Saccharomyces*	Peng and Yu (2011)
Fuzhuan tea	*Aspergillus, Penicillium*	Zhang et al. (2010)
Fuzhuan tea	*Aspergillus, Penicillium*	Zhao H. et al. (2021)
Fuzhuan tea	*Aspergillus*	Ruan (2015)
Fuzhuan tea	*Aspergillus, Penicillium*	Hu (2012)
Fuzhuan tea	*Aspergillus, Penicillium*	Liu et al. (2011)
Fuzhuan tea	*Aspergillus*	Yang et al. (2020)
Fuzhuan tea	*Aspergillus, Penicillium*	Wu et al. (2021)
Liupao tea	*Aspergillus, Penicillium*	Wen et al. (2012)
Liupao tea	*Aspergillus*	Chen R. et al. (2016)
Liupao tea	*Aspergillus*	Ou et al. (2017)

As can be seen from Table 4, fungi of the genera *Aspergillus* and *Penicillium* play a very significant role in the processing of dark tea. There has been a lot of research on the dominant microorganisms of different types of dark tea, but there has been little research on whether these dominant microorganisms can produce fungus-derived toxins.

### 3.1. Aspergillus

Many *Aspergillus* spp. fungi are the dominant species in the production and processing of dark tea and have a strong relationship with the formation of dark tea quality (Jiang, 2012a; Wu J., 2013; Xiong, 2017). At present, only *Eurotium cristatum* has been specified by the Chinese national standard as the physicochemical index of Fuzhuan tea.

There are parts of fungi in the genus *Aspergillus* that produce mycotoxins, such as *Aspergillus flavus*, *Aspergillus fumigatus*, *Aspergillus niger*, and *Aspergillus versicolor*.

During the fermentation of dark tea, the number of *Aspergillus* spp. is always in a dominant position. The fungi *Aspergillus* spp. have an essential contribution to the quality formation of dark tea. For example, *Aspergillus niger* metabolism can produce organic acids and enzymes that have a variety of hydrolytic enzymes, including oxidases, glycosidases, and proteases, which can hydrolyze polysaccharides, fats, proteins, and cellulose into monosaccharides, amino acids, and some soluble carbohydrates, so that the biochemical components in the tea can be easily leached out and enhance the thickness of the tea broth. *Aspergillus niger* is a comparatively safe industrial fungus that was recognized worldwide in the 20th century and is a commonly used industrial fermentation strain. However, in recent years, there have been plenty of studies finding that some strains of *Aspergillus niger* could produce mycotoxins. Although only a few *Aspergillus niger* fungi are able to produce mycotoxins, we absolutely need to pay more attention to them.

### 3.2. Penicillium

Fungi of the genus *Penicillium* are also frequently detected during dark tea processing and in finished dark tea (Haas et al., 2013). It is widely used in the processing and storage of dark tea production.

There are more than 600 species of fungi in the genus *Penicillium*, and their morphological characteristics are generally similar to each other. Many researchers have also isolated and identified *Penicillium* spp. fungi from dark tea, as shown in Table 5, and *Penicillium* spp. fungi that may produce fungal toxins are shown in Table 6.

**Table 5:** Common *Aspergillus* spp. and *Penicillium* spp. fungi in dark tea.

Type of dark tea	Species	References
Ya’an Tibetan tea	*Aspergillus niger, Aspergillus fumigatus, Aspergillus miscellaneous, Penicillium citrinum*	Li (2019)
Fuzhuan tea during ‘flowering’	*Aspergillus niger*, *Aspergillus fumigatus*, *Aspergillus confusus*	Zhao (2012)
Brick tea	*Aspergillus pseudogalactiae*	Wang et al. (2015)
Fuzhuan tea	*Aspergillus costiformis Kong& Qi, Aspergillus niger and Aspergillus oryzae*	Wu et al. (2021)
Fuzhuan tea	*Aspergillus sojae, Penicillium purpurogenum, Penicillium chrysogenum*	Liu et al. (2011)
Liupao tea	*Aspergillus chevalieri*, *Aspergillus restrictus*, *Aspergillus tubingensis*,	Chen R. et al. (2016)
Liupao tea	*Aspergillus niger, Aspergillus tubingensis, Aspergillus fumigatus, Aspergillus oryzae, Aspergillus sydowii, Aspergillus ochraceus, Aspergillus tamarii and Aspergillus sloerotiorum, Penicillium citrinum*, *Penicillium chrysogenum*, *Penicillium oxalicum*, *Penicillium chermesinum*, *Penicillium meleagrinum*	Xu (2014)
Sichuan dark tea	*Aspergillus niger, Penicillium citrinum, Penicillium crustosum, Penicillium brevicompactum, penicillium georgiense, penicillium brocae*	Xiong (2017)
Liupao tea	*Penicillium jiangxiense*	Ou et al. (2017)
Dark tea	*Aspergillus niger, Aspergillus tabinum, Aspergillus carbonarius, Aspergillus nidulans, Aspergillus ochraceus, Penicillium verrucosum*	Zhao Z. et al. (2021)

**Table 6:** *Penicillium* fungi that may produce fungal toxins in dark tea.

Fungi	Fungal toxin of possible production
*Penicillium citrinum*	Citrinin (Guo et al., 2019)
*Penicillium chrysogenum*	Citrinin; Patulin (Zhou et al., 2015a)
*Penicillium purpurogenum*	Citrinin (Chai et al., 2012)
*Penicillium viridicatum*	Ochratoxin A; Citrinin (Pitt, 1987)
*Penicillium verrucosum*	Ochratoxin A (Zhao Z. et al., 2021); Citrinin (Bragulat et al., 2008)

### 3.3. Other genus

Li (2019) isolated fungi of the genus *Rhizoctonia* from Ya’an Tibetan Tea. Zhao (2012) isolated one strain of *Fusarium* spp., one strain of *Trichoderma* and three strains of *Mucor* spp. from the ‘flowering’ processing of Fuzhuan tea. Wu et al. (2021) isolated *Fusarium equiseti*, *Alternaria*, and *Cladosporium cladosporioides* from Fuzhuan tea. Wen et al. (2012) isolated *Mucor* spp. and *Rhizoctonia* spp. from Liupao tea fermentation samples. All of the above fungi have the ability to produce mycotoxins, but few researchers have paid attention to them previously. These are the questions and directions that we need to pay close attention to in the future, whether these fungi can produce mycotoxins on tea substrates or not.

These fungal genera that have been isolated and identified as having the potential to cause adverse health effects to consumers are *Fusarium*, *Xylaria*, *Streptomyces*, and, *Trichoderma* spp. The fungi of the genus *Fusarium* mainly include *Fusarium graminearum*, *Fusarium verticillioides*, *Fusarium nivale*, *Fusarium tricinctum*, and *Fusarium equiseti* etc. (Zu et al., 2021). These fungi may produce toxic secondary metabolites such as trichothecenes, zearalenone, moniliformin, and butenolide (Huang et al., 2017). Some strains of *Trichoderma* such as *T. reesei* and *T. viride* are capable of producing gliotoxin, which belongs to the Tricothecenes (Mao and Zhang, 2018). Some strains of *Alternaria* can produce a variety of mycotoxins such as alternariol (AOH), alternariolmethylether (AME), and tenuazonicacid (TeA) (López et al., 2016). *Mucor* spp. is also a pathogenic fungus, often causing mold in food, Mucor can enter the body through the respiratory tract, digestive tract, or skin, causing blood clots and tissue necrosis (Yang and Liu, 2021).

In summary, many researchers have isolated and identified the microorganisms from dark tea. Therefore, a plentiful toxin-producing source has been discovered. The isolation and identification of toxin-producing fungi in dark tea require us to think highly of the safety of dominant fungi in the processing of dark tea and avoid the infestation of toxicity-producing fungi. We need to pay further attention to whether these fungi produce mycotoxins and whether they can cause health effects in consumers. Only a small number of researchers have paid attention to the toxicity-producing properties and isolating conditions of dark tea. However, in order to ensure the safety of dark tea production, research on the infestation of harmful fungi in dark tea is unquestionably required.

## 4. Possible contamination pathway

**Figure 2:**
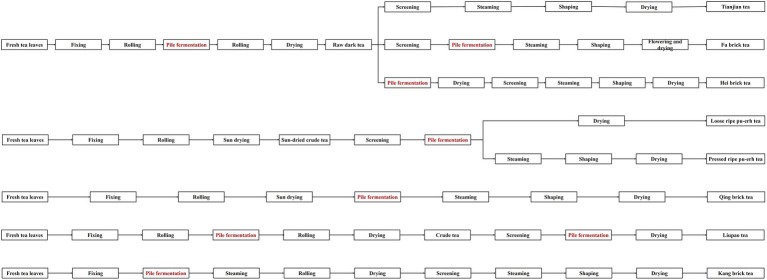
The main manufacture processing of various dark tea.

The initial production process of dark tea can be roughly divided into fixing, rolling, pile fermentation, and drying. Dark tea can be mainly classified into Hunan Fuzhuan tea, Sichuan Kangzhuan tea, Hubei Qingzhuan tea, Guangxi Liupao tea, Yunnan ripe Pu-erh tea, and Shaanxi Fuzhuan tea, etc., and their main manufacture processes are shown in Figure 2.

Each origin of dark tea has its own unique processing process. Fermentation and storage are the main ways for dark tea to be infested by harmful fungi, and if it is infested by toxicity-producing fungi during processing, it is likely to lead to the contamination of dark tea with fungal toxins.

### 4.1. Pile fermentation

The pile fermenting process is crucial in defining the quality of dark tea. The essence of fermentation is the enzymatic reaction of extracellular enzymes produced by the secretion of dominant microorganisms and the action of moist heat (heat of microbial respiration and metabolism) combined.

A series of complex transformations occur to form the unique flavor quality of dark tea (Wang et al., 1991a). During processing, high temperature and high humidity conditions are favorable for microbial growth and the metabolic transformation of black tea, but if the dark tea is contaminated with harmful fungi during the pile fermentation, then there is a risk that the dark tea will be contaminated with fungal toxins.

The microorganisms in the fermentation process are the more abundant stage in the whole dark tea production process (Zhang et al., 2017; Xu Z. et al., 2019). Wen et al. (2012) isolated *Aspergillus niger*, *Aspergillus oryzae*, and *Aspergillus glaucus* from Liupao tea fermentation samples. Wang Q. et al. (2020) and Dong et al. (2009) isolated *Aspergillus niger*, *Aspergillus tamarii*, *Aspergillus fumigatus*, *Aspergillus clavatus*, *Aspergillus oryzae*, *Aspergillus glaucus*, *Aspergillus terreus*, *Aspergillus candidus*, *Aspergillus wentii* var. *fumeus*, *Aspergillus penicillioides*, *Aspergillus aureolatus*, *Aspergillus egyptiacus*, *Aspergillus foetidus*, *Aspergillus japonicus Saito var*. *japonicus*, *Aspergillus restrictus Smith*, etc., from the fermentation process of Pu-erh tea.

### 4.2. Storage

Dark tea is a kind of post-fermented tea with the characteristic that “flavor improves with ages,” and its flavor is unique. The change of the inner material component of dark tea is intimately related to temperature, humidity, and age (Chen Y. et al., 2016). Therefore, during the storage process, dark tea is always changing silently, and the degree is accountable for the storage conditions. Mildew and deterioration of the dark tea are brought on by improper storage, thus leading to the contamination of dark tea by fungal toxins.

Dark tea during storage is probably infested with harmful fungi due to high humidity conditions and thus. Xu et al. (2016) investigated mycotoxin residues under natural moldy and exogenous inoculation with toxin-producing *Aspergillus flavus* conditions by artificial high humidity mold-promoting culture, and all three mycotoxins were not detected in naturally moldy dark tea samples, while AFB_1_ was detected in tea samples with exogenous inoculation with *Aspergillus flavus* strains. Zhong et al. (2010) isolated and identified a total of one *Aspergillus* strain, three *Penicillium* strains, and one *Rhizopus* strain for the fungal populations present in the storage of Ya’an Tibetan tea. Zhou et al. (2015a,b) isolated and identified a strain of *Aspergillus niger* from aged Pu-erh tea for solid-state fermentation of Pu-erh tea, and OTA was detected in the fermented Pu-erh tea samples, which proved that this strain of *Aspergillus niger* has the ability to produce OTA. In Li’s finding, total of 218 fungi was isolated from Liupao tea, and some of these strains, such as *Aspergillus ochraceus*, *Aspergillus oryzae*, *Penicillium citrinum*, and *Penicillium chrysogenum* can produce CIT (Li et al., 2020). Zhao et al. (2021) isolated 560 fungal strains from dark tea samples, including seven species of *Penicillium* spp. and 13 species of *Aspergillus* spp., and 20 species of other genera. Six different species of isolated fungi were identified had the ability to produce OTA. Nearly all strains belonging to species of *Aspergillus carbonarius*, *Aspergillus ochraceus*, *Aspergillus nidulans* and *Penicillium verrucosum* were capable of producing OTA, but only part of *Aspergillus niger* and *Aspergillus tubingensis* can produce OTA. Zhao et al. (2022) isolated 18 strains of *Aspergillus* spp. and *Penicillium* spp. fungi from dark tea samples of different years, and one *Aspergillus niger* was found to have the ability to produce OTA. Although dark tea has the characteristic of “flavor improves with ages,” if it is not stored properly, it may becomeinfested with harmful fungi and thus become moldy or produce fungal toxins that are harmful to human health.

The possibility of dark tea being contaminated by fungi, which warns that dark tea enterprises need to concentrate on the contamination of harmful microorganisms during the production process. It’s necessary to constantly pay attention to the changes in environmental conditions during the storage of dark tea to prevent it from being contaminated by fungi during the storage process. The standardized of the production of dark tea is urgently needed to ensure the safety of the dark tea production, processing, and storage environments, which is the guarantee for the prosperous development of the dark tea industry.

## 5. Discussion

Dark tea is potentially contaminated with toxin-producing fungi (Jiang et al., 2018; Deng et al., 2020). Microorganisms in dark tea play an important part in the formation of dark tea sensory quality, while some microorganisms can lead to tea fungal toxin contamination (Xin and Liang, 2020). During the processing of dark tea, extracellular enzyme catalysis by microorganisms is the dominant factor, thus promoting the transformation of dark tea quality and forming its unique flavor. Existing microbial studies on dark tea have identified *Aspergillus* spp. as the dominant fungal group in the fermentation process of dark tea. However, from the perspective of food safety, *Aspergillus* spp. fungi can produce a variety of fungal toxins, such as: aflatoxin, ochratoxin, citrinin, and vomitoxin (Sengun et al., 2008; Bräse et al., 2009; Zhao et al., 2017; Li et al., 2021). In recent years, the debate on whether dark tea contaminated with harmful fungi can produce fungal toxins has become more intense. Public opinion often emerges that tea contains fungal contaminants that can cause cancer, and some scholars have also reported studies on dark tea being contaminated by fungal toxins (Liu et al., 2016, 2017; Cui et al., 2020; Li et al., 2020; Zhao et al., 2022), which has had a huge impact on the development of the industry.

Dark tea has increased the risk of mycotoxins contamination due to the involvement of fungi during the microbial and hygrothermal dominated solid-state fermentation process. Zhou et al. (2015a,b), Zhang et al. (2016), and Zhao Z. et al. (2021) have isolated toxin-producing fungi from dark tea, but the detection rate and content of mycotoxins in tea were far below expectations. Zhao et al. (2015) suggested that in the solid-state fermentation process, there is a possibility of the presence of antagonistic microorganisms that inhibit the growth of harmful fungi and the production of mycotoxins. The results of some other studies have shown that certain endogenous components in tea are capable of inhibiting fungal toxin production (Wu Q., 2013; Li H. et al., 2015; Guo, 2018).

At the same time, the tea matrix composition is highly complex and specific, containing abundant tea pigments, polyphenols, caffeine, and other substances, which are the material basis for the health functions of tea, and such secondary metabolites also have the ability to stop or inhibit mycotoxin production by mycorrhizal fungi in the presence of fungal contamination (Zhao et al., 2017; Jiang et al., 2018). In a study of aflatoxin production by *Aspergillus flavus* using tea substrates, results showed that aqueous extracts from Yunnan Big Leaf tea had a significant inhibitory effect on the synthesis of aflatoxins, they speculated that the tea extract may have inhibited aflatoxin synthesis by modulating the expression of *aflR* (Li H. et al., 2015). In recent years, it has also been found that there is a close relationship between aflatoxin biosynthesis and oxidative stress, and that the presence of antioxidants inhibits the synthesis of AFB_1_ (Wu Q., 2013; Zhao, 2014; Guo, 2018). All these studies show that the active substances in tea have the ability to inhibit the production of toxins from mycotoxins, but there is a limit to the inhibitory effect of active substances on harmful fungi, and what we need is to completely and effectively avoid contamination of tea with harmful mycotoxins to effectively and efficiently protect the safety of consumers.

There are many studies on mycotoxins in dark tea, and most of the findings suggest that the level of mycotoxin exposure in dark tea is insufficient to threaten the health of tea consumers (Liu et al., 2016; Mo et al., 2016; Hu et al., 2019). The results of mycotoxin detection in dark tea are inconsistent due to different sources of dark tea samples as well as different methods of determination. Results of ELISA were mostly positive for mycotoxins (Santos et al., 2009; Li W. et al., 2015), while LC–MS/MS results were mostly negative, with only a few positive cases present. The possible reasons for this situation are due to the results for ELISA are probably false positive, and LC–MS/MS results seem more reliable. The complexity of the tea matrix, the conversion of polyphenols to pigment substances such as theaflavins and thearubigins during the fermentation of dark tea by the action of moist heat and microbial extracellular enzymes (Jiang, 2012a; Zhang, 2015; Ji et al., 2016), which can affect the results of ELISA.

If the sample pretreatment method is not appropriate and the interferences, such as tea pigment substances are not removed, the ELISA or HPLC assay will easily show false positives due to the interference of pigment substances. Another possible reason is that the mycotoxins in tea transform themselves or combine with certain substances in tea to form masked mycotoxins that are difficult to detect. At present, the reliable mycotoxin detection methods commonly used in dark tea products are high performance HPLC and HPLC-MS, but if the mycotoxin forms are masked by certain substances in dark tea under specific conditions, it is difficult to be detected by the above two methods. Due to the complexity of the tea matrix and the instability of certain mycotoxins, the formation of masked mycotoxins in dark tea also has a greater possibility.

So far, there are more studies on the exposure of mycotoxins in tea, but there are fewer studies on the exposure, toxicity, and contamination status of masked mycotoxins. The results of some reports have shown that some of the masked mycotoxins may re-release their prototypes after entering the digestive systems of humans and animals, posing serious potential safety hazards to food. The toxicity of many masked mycotoxins may be much lower than their prototypes or even non-toxic, but if they react again in the human body, then there will be a greater safety risk. Therefore, it is necessary to conduct basic research on the formation, regulatory mechanisms, contamination status, and detection methods of the major masked mycotoxins in tea to provide a scientific basis for effective prevention, control, and safety supervision of masked mycotoxins in tea.

The most effective way to prevent mycotoxin contamination in dark tea is to avoid the infestation of harmful microorganisms during production, processing, and storage, as well as to eliminate mycotoxin production from the source. It is urgent to perform a precise screening of beneficial microorganisms and avoid harmful microbial infestation, which is also an effective way to prevent fungal toxin contamination in dark tea. It is also the direction of future efforts by dark tea producers.

## Author contributions

WX and YZ: conceptualization, investigation, draft reviewing, and editing. Y-qZ: conceptualization, investigation, data curation, writing – original draft preparation, and writing – review and editing. W-bJ: conceptualization, investigation, and data curation. S-yL: conceptualization and investigation. TB: draft reviewing, revision, and editing. All authors contributed to the article and approved the submitted version.

## Funding

This work was supported by Sichuan Province S&T Project (2021ZHFP0021, 2022ZHXC0022, 2023YFH0025, and 2023YFN0010) and Ya’an Yucheng District School Cooperation Project (2022).

## Conflict of interest

The authors declare that the research was conducted in the absence of any commercial or financial relationships that could be construed as a potential conflict of interest.

## Publisher’s note

All claims expressed in this article are solely those of the authors and do not necessarily represent those of their affiliated organizations, or those of the publisher, the editors and the reviewers. Any product that may be evaluated in this article, or claim that may be made by its manufacturer, is not guaranteed or endorsed by the publisher.
